# Whole exome sequencing of 28 families of Danish descent reveals novel candidate genes and pathways in developmental dysplasia of the hip

**DOI:** 10.1007/s00438-022-01980-5

**Published:** 2022-12-01

**Authors:** Maja Dembic, Lars van Brakel Andersen, Martin Jakob Larsen, Inger Mechlenburg, Kjeld Søballe, Jens Michael Hertz

**Affiliations:** 1 Department of Clinical Genetics, Odense University Hospital, J. B. Winsløws Vej 4, 5000 Odense C, Denmark; 2 Department of Mathematics and Computer Science (IMADA), University of Southern Denmark, Campusvej 55, 5230 Odense M, Denmark; 3 Department of Clinical Research, University of Southern Denmark, Winsløwparken 19, 5000 Odense C, Denmark; 4 Department of Orthopedic Surgery, Aarhus University Hospital, Palle Juul-Jensens Boulevard 99, 8200 Aarhus N, Denmark; 5 Department of Clinical Medicine, Aarhus University, Palle Juul-Jensens Boulevard 82, 8200 Aarhus N, Denmark

**Keywords:** DDH, Whole exome sequencing, DNA mutation, Detection of mechanical stimulus

## Abstract

Developmental dysplasia of the hip (DDH) is a common condition involving instability of the hip with multifactorial etiology. Early diagnosis and treatment are critical as undetected DDH is an important cause of long-term hip complications. Better diagnostics may be achieved through genetic methods, especially for patients with positive family history. Several candidate genes have been reported but the exact molecular etiology of the disease is yet unknown. In the present study, we performed whole exome sequencing of DDH patients from 28 families with at least two affected first-degree relatives. Four genes previously not associated with DDH (*METTL21B*, *DIS3L2*, *PPP6R2*, and *TM4SF19*) were identified with the same variants shared among affected family members, in more than two families. Among known association genes, we found damaging variants in *DACH1*, *MYH10*, *NOTCH2, TBX4, EVC2*, *OTOG*, and *SHC3*. Mutational burden analysis across the families identified 322 candidate genes, and enriched pathways include the extracellular matrix, cytoskeleton, ion-binding, and detection of mechanical stimulus. Taken altogether, our data suggest a polygenic mode of inheritance for DDH, and we propose that an impaired transduction of the mechanical stimulus is involved in the etiopathological mechanism. Our findings refine our current understanding of candidate causal genes in DDH, and provide a foundation for downstream functional studies.

**Supplementary Information:**

The online version contains supplementary material available at 10.1007/s00438-022-01980-5.

## Introduction

Developmental dysplasia of the hip (DDH, OMIM #142700) is a common condition characterized by abnormal hip development during infancy and early development, with a wide spectrum of phenotypic presentations (Yang et al. 2019). Clinical diagnosis of DDH in older children presents challenges as early features are non-specific, and the disorder might be at an advanced stage by the time of diagnosis. However, early diagnosis and treatment is critical to achieve the best possible outcome. Early treatment consists in obtaining a stable hip joint with minimal complications as early as possible, which is usually achieved with a brace that secures the hip in a flexed and abducted position (Dezateux and Rosendahl 2007). Undetected hip dysplasia increases the risk of long-term complications, including pain, secondary osteoarthritis, and surgery (Dezateux and Rosendahl 2007; Harsanyi et al. 2020b).

The global incidence of DDH is estimated to be between 0.1 and 6.6 per 1000 live births and is subject to ethnic variation, with the highest prevalence among Japanese, Native American, and European populations (Dezateux and Rosendahl 2007; Harsanyi et al. 2020b). The etiology of DDH is multifactorial including genetic, mechanical, and environmental factors. Some of the risk factors that increase the occurrence of DDH include: female gender, breech position at birth, fetal position, oligohydramnios, a large birth weight, primiparity, and swaddling (Harsanyi et al. 2020b). The influence of genetics in DDH is well-established from studies on families with multiple individuals affected by DDH (Basit et al. 2016; Gkiatas et al. 2019), and monozygotic twins (Geiser et al. 1959). Multiple genes have been related to the disorder. These are mainly genes coding for connective tissue proteins, genes regulating osteogenesis and chondrogenesis, formation of joint structures, and chemokine receptors (Harsanyi et al. 2020b). An autosomal dominant mode of inheritance with incomplete penetrance is most often considered for DDH, although some studies proposed a multiple gene inheritance (Wynne-Davies 1970; Basit et al. 2017). Given the phenotypic and genotypic complexity of DDH, it is possible that DDH is caused by a combined action of multiple genes.

The etiopathogenesis of DDH has not been fully elucidated, and more high-throughput genetic studies are needed to resolve the issue of heritability, which would lead to better preventive strategies, especially in newborns with positive family history. Here we performed a comprehensive genetic analysis on 29 families where at least two relatives were affected by DDH. We investigated single nucleotide variants (SNVs), inserts and deletions (indels), and copy number variations (CNV) within single families and we performed a mutational burden analysis across the families. We used protein interaction and pathway analysis to identify gene sets associated with DDH, allowing us to gain insights into the mechanism of the disease. We identified novel candidate genes, confirmed other known association genes, and proposed a biological pathway involved in the etiopathogenesis of the disease.

## Materials and methods

### General overview of the project

We carried out genetic analysis of 66 affected individuals of Danish origin, from a total of 29 families, with confirmed DDH diagnosis through radiological and clinical examination, ascertained through the Danish periacetabular osteotomy (PAO) Database at Aarhus University Hospital (Simonsen et al. 2019). Our study included only affected individuals within the families; as a strategy, it may certainly have advantages: as DDH is known for incomplete penetrance and including healthy relatives as controls may have confounding effects. As controls we used minor allele frequencies (MAFs) of each variant in the total population according to the Genome Aggregation Database (gnomAD) database v2.1.1 (GRCh37/hg19), previously known as the Exome Aggregation Consortium (ExAC). As population-specific controls we used a database with reported frequencies from a study including 2000 Danish diabetic patients (D2000) sequenced by whole exome sequencing (Lohmueller et al. 2013).

### Whole exome sequencing

After obtaining written informed consent, we collected blood EDTA samples and performed whole exome sequencing (WES). Genomic DNA was isolated using standard methods. The libraries were prepared on a Beckman Coulter Biomek 4000 Laboratory Automation Workstation using Roche NimbleGen SeqCap EZ MedExome Enrichment Kits. Targeted 2 × 75 bp pair end sequencing was performed on an Illumina NextSeq 550 platform.

### Data analysis

Raw reads were processed using the Burrows‐Wheeler Alignment tool (BWA‐MEM) v. 0.7.12, and aligned to reference sequence (human NCBI build 37). GATK Best Practice pipeline v.4.1.4.1 was used for variant calling (Van der Auwera et al. 2013). MarkDuplicates (Picard Toolkit) was used to remove duplicates. Base quality score recalibration was done using BaseRecalibrator and ApplyBQSR, post-recalibration BAM files obtained as output were used in the final analysis. After data processing, Picard (CollectMultipleMetrics) was used to check the mapping quality and gather information regarding GC content, insert size, coverage, percentage of aligned bases, general error rate, and duplication rate. HaplotypeCaller was used to call germline SNPs and indels.

### Kinship, principal component, variant calling quality control, and statistical analysis

After the variant calling files (vcf) were obtained, they were merged and uploaded into PLINK 2.0 software. Variants with a variant allele frequency of under 0.05 were removed. Population stratification was analyzed by using the principal component analysis (PCA) function. Ten principal components were extracted and eigenval and eigenvec values uploaded into R software to make the PCA plot. KING—robust kinship estimator in PLINK 2.0 was used to calculate the relatedness between the samples in a pairwise manner. Obtained kinship coefficients were plotted against the identity-by-state (IBS0) values to visualize the relatedness between the samples.

Picard (CollectVariantMetrics) was used to gather quality metrics on the called variants and measure transition to transversion (Ti/Tv) ratios. Ti/Tv is an indicator of sequencing quality. The ratio varies by genome region, genome functionality, and to a lesser extent by ancestry. In sequencing data Ti/Tv is generally under 3.0, and above 2.0 outside exome regions (Wang et al. 2015). Heterozygosity was measured as the ratio between the number of heterozygote and homozygote SNPs obtained from individual vcf files, after removal of low-quality variants (presenting more than 2 alleles) and all indels.

### Variant annotation and selection

VarSeq software (Golden Helix) was used for variant annotation and selection, and it was done through a cascade of filtering steps. The following quality cut-offs were used: (1) accuracy base call of at least 99% (Q20), and (2) variant allele frequency (VAF) in reads ≥ 25%. The variants were also checked manually at a later stage and variants located in multimapped regions or regions with high background noise and low mean quality (< 20) in BAM files were excluded. We excluded as well variants with less than 20X coverage, or originating from alignment or PCR mistakes. To select for DDH-specific, rare, and functional variants in single families, we filtered coding and splicing variants through the following pipeline: (1) presence in all affected individuals in the family, (2) MAF ≤ 0.01 (gnomAD database), (3) and less than 5 homozygotes in the gnomAD database. Splicing region variants, residing outside of the GT-AG dinucleotide, were assessed by Alamut Visual v.2.8 software and included only when predicted to significantly affect splicing. We used VarSeq for CNV calling and analysis. We selected CNVs that cover at least four exons, occurred in all affected individuals of a family, and presented with low-frequency rates in the gnomAD database and the database of the genomic variants (DGV) (≤ 0.02).

As a population-specific control we used variant frequencies from a published database of exome data from 2000 Danish individuals (Lohmueller et al. 2013). A one-sided Fischer’s Exact Test was used to check whether the variants were enriched (over-represented) in our sample compared to the Danish control sample. The test was carried out in R software after constructing a contingency table with the number of variant alleles in each study group in comparison, and the total number of alleles in each study group. A *p-*value of 0.05 was used as cut-off for significantly enriched variants in our study group.

The correlation test was done in R software using the length of each gene containing the 5’ and the 3’ UTR regions, and the coding sequence (cds) as downloaded from BioMart Ensembl. The correlation was tested between length and the number of variants found in each gene, and length and the number of families carrying variants in a gene.

### Functional analysis of variants

To select for the most damaging and DDH-related variants we further filtered the variants keeping only those with less than 590 alternate allele counts (the expected allele count assuming an autosomal dominant mode of inheritance and given the average DDH incidence of 1:3400 (Harsanyi et al. 2020b)) in gnomAD. We used the Combined Annotation Dependent Depletion (CADD Scores 1.4) method to score the variants for deleteriousness, PHRED scaled scores were used (Rentzsch et al. 2019). The CADD algorithm includes conservation metrics, functional genomic data, exon–intron boundaries, and protein functionality scores. A score equal or greater of 20 indicates that a variant is predicted to be among the 1% of the most deleterious substitutions. We used a CADD score of over 25 to select for the most damaging variants in our study group. We further used a dbNSFP tool (Kircher et al. 2014) that incorporates six different algorithms to evaluate the functional impact of a variant, including: SIFT, Polyphen, MutTaster, MutAssessor, FATHMM Pred, and FATHMM MKL. The most damaging variants were selected based on a voting system where five or all six of these algorithms predicted a negative functional impact. To estimate which variants were more likely to be involved in DDH we used the PhoRank algorithm and hip dysplasia as user-specified phenotype. PhoRank assigns a score to each gene based on their proximity to a user-specified phenotype, using Gene Ontology (GO) and Human Phenotype Ontology (HPO)(Singleton et al. 2014). The genes are ranked from 0 to 1 where 1 indicates the closest direct, or through a shared gene, relationship.

### Genetic overlap, GO term, pathway, and STRING analysis

We created an R-script to make a list of all the genes and variants we found in the families and to filter for those that occur in multiple families. For term and pathway enrichment analysis, we used the list of 322 genes with variants in multiple families and the following databases (STRING v.11.5, https://string-db.org/). Gene Ontology (GO) (http://geneontology.org/) is a comprehensive database of current knowledge of biological systems, where molecular function, biological process, and cellular component are the three aspects used for classification of the genes. Kyoto Encyclopedia of Genes and Genomes (KEGG) (https://www.genome.jp/kegg/pathway.html) is a collection of maps representing known molecular interactions and networks, while Reactome (https://reactome.org/) is a pathway database. The enrichments are calculated as strength measured with the formula: Log10 (observed/expected). Only terms with a false discovery rate (FDR) of under 0.05 are reported. They were calculated using the Benjamini–Hochberg procedure for multiple testing correction within each category. The protein interaction network was analyzed and visualized using STRING. STRING is a database of physical and functional, known and predicted, protein–protein interactions (Szklarczyk et al. 2019). The STRING online resource performs network and enrichment analysis, visualizes protein networks, and reports a statistical analysis of enrichments of connections between proteins, and functional subsystems.

## Results

### Sample collection and clinical description

After obtaining written informed consent, we collected blood samples from 66 patients (54 (82%) females and 12 (18%) males, all of Danish descent) diagnosed with DDH, belonging to a total of 29 families. Each of the families had at least two affected individuals. One family had five affected first-degree relatives, five families had three affected first-degree relatives, while the remaining 23 families had two affected first-degree relatives (the pedigrees are shown in Online Resource 1).

### Whole exome sequencing

**Fig. 1:**
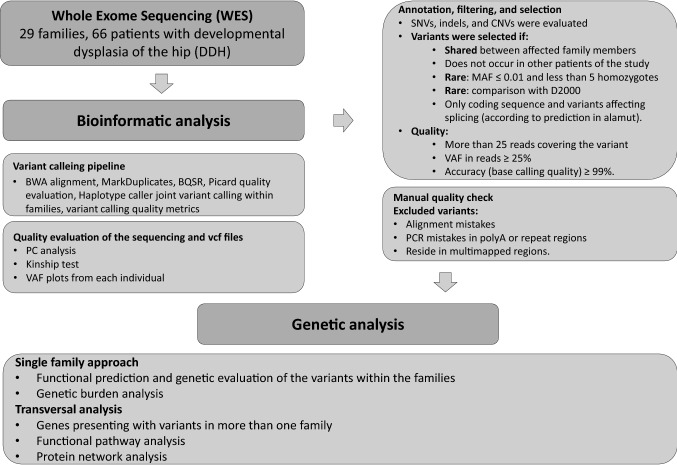
General overview of the project**.** We performed whole exome sequencing of 66 patients from 29 families. After alignment and variant calling, the variants were filtered according to frequency and quality, and were manually checked for artefacts. We undertook a single family approach performing genetic analysis of the variants within each of the family, and a transversal approach across families identifying genes occurring with variants in more than one family. Abbreviations: CNVs: copy number variations, D2000: a database of 2000 Danish individuals, GO: Gene Ontology, InDels: inserts and deletions, MAF: minor allele frequency population wise, SNVs: single nucleotide variants, UTR: untranslated region, VAF*:* variant allele frequency (in reads)

To investigate the possibility of a shared genetic cause of DDH in our cohort, we performed WESof the affected individuals. A study structure diagram is shown in Fig. 1.

Variant allele frequencies in the reads (VAF) were plotted for each sample to check whether there are cross-contaminations (Supplementary Fig. S1 in Online Resource 2). Peaks in values between 0, 0.5, and 1 would suggest presence of variants from another sample or other quality issues. Our samples presented VAF plots that do not indicate cross-contamination. The plot for a sample in family 28 suggested quality issues.

Further evaluations of the sequencing quality showed no issues in coverage, sequencing depth, and variant calling process for most of the samples. The mean sequencing coverage was 140.5X, while on average 94.2% of the target regions had a sequencing depth of at least 30X (further details are provided in Online Resource 3). As quality control measurement, we evaluated the transition (Ti) to transversion (Tv) ratio and the heterozygosity ratio, commonly used as quality control parameters in high-throughput sequencing data. We found an average Ti/TV ratio of 2.73 (± X), which is within the normal values for the European ancestry population in exome data (Wang et al. 2015), (Online Resource 3, variant calling metrics Ti/TV and heterozygosity ratios tabs). While average heterozygosity values were 1.63 ± X, slightly above for what reported for Finnish, British, Scottish and Iberian populations (Samuels et al. 2016). Sample A5B3 from family 28 had discordant data from the rest, indicating poor sequencing quality, and, therefore, it was excluded from subsequent analysis.

Next, we did a relatedness check to confirm the correct assignment of the samples within the families (Supplementary Fig. S2 in Online Resource 2). This check confirmed that samples where correctly placed and joint-variant calling within families was correctly performed. PCA was done to check the homogenous composition of our study group, as it would be expected if all individuals were of Danish ancestry. Family 34 with 5 members showed to be distanced from all the other individuals on the first component axes (Supplementary Fig. S3 in Online Resource 2), suggesting a different population ancestry from the rest. Therefore, we also excluded family 34 from further analysis. We next proceeded with annotation of the variants and genetic analysis within and across families. We searched for SNVs, indels, and CNVs shared within the families. After alignment and joint variant calling within each family, the resulting variant calling files (vcf) contained an average of 49,737 variants (Online Resource 3). We further filtered to include only high quality, coding sequence, and splicing variants, with a VAF in reads of 25% or more. As control reference, we used variant frequencies in the general population from gnomAD, which is based on data from 125,748 exomes and 15,708 whole-genomes from unrelated individuals situated worldwide. We kept only the variants with a (MAF equal or under 0.01, and with less than five reported homozygotes in the overall global population. We kept only shared variants between affected members within the families, and excluded any variant present in other families, but not shared within all the members. After obtaining a list of all the variants, we compared them to the frequencies found in 2000 Danish diabetic patients using Fischer’s Exact Test as an additional population control. We kept only the variants that had a sufficiently greater frequency in our sample, compared to the Danish control sample. Frequency and *p*-values for each variant are listed in Online Resource 4.

### Variant distribution and annotation

We found a total of 2640 rare and shared variants in a total of 2276 genes, carried by related individuals across the 28 families (the whole list of genes and variants is given in Online Resource 4). The great majority of the variants were missense (2379), followed by a relatively small number of stop-gain variants (64), splicing mutations (61), and frameshift variants (67) (further details on variant type distribution are given in Online Resource 5).

**Fig. 2:**
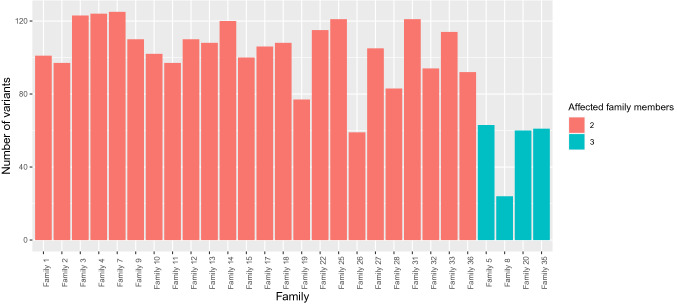
Distribution of the variants across the families**.** In the figure is shown a histogram representing the distribution of 2640 rare variants across the families. These included all splicing and coding sequence variants, with a MAF ≤ 0.01, and shared between the affected individuals within a family. Most of the families had only two members, four families had three members

In Fig. 2 is shown the number of variants identified in each family. On average, two-member families shared 104 rare variants. As expected, families with three members shared a smaller number of variants (52) than two-member families, suggesting a certain amount of false positives. In family 8 the lowest number of shared variants was found (24), due to the fact of three family members analyzed including a second and a third degree relative.

In Table 1 are listed the rare variants that have been found shared among affected family members in more than one family. Only two variants are predicted to be damaging: *METTL21B*: NM_015433.2:c.620G > A, and *PPP6R2*: NM_001242898.1:c.1421G > A. *METTL21B* encodes for a Lysine Methyltransferase, while the *PPP6R2* gene encodes a regulatory subunit of the serine/threonine protein phosphatase 6 catalytic subunit (PPP6C). None of them have been previously described in relation to DDH.

**Table 1:** Variants occurring in multiple families. The table shows a list of rare variants that have been detected in multiple families. All of the variants are missense; the functional prediction for deleteriousness is listed next to them. A CADD score of over 20 indicates that a variant is among the 1% most deleterious substitution in the genome, and thus is considered damaging. Additionally, we used other six tools for evaluating a negative functional impact (SIFT, Polyphen, MutTaster, MutAssessor, FATHMM Pred, and FATHMM MKL). The results of a voting system from these six algorithms is shown. A higher number of tools predicting a variant damaging gives a higher score in the voting system. Only two variants have an increased deleteriousness score: *METTL21B*: NM_015433.2:c.620G>A, and *PPP6R2*: NM_001242898.1:c.1421G>A. Abbreviations: CADD: combined annotation dependent depletion; PPP: phosphoprotein phosphatases; SNP: single nucleotide polymorphism, AF: allele frequency; D2000: database of variants found in 2000 Danish diabetic patients

Gene	Variant	Protein	Type	Consequence	AF GnomAD exomes	AF D2000	Family	CADD score	Number of tools predicting damaging
*METTL21B*	NM_015433.2:c.620G > A	NP_056248.2:p.Arg207Gln	SNP	Missense	0.0018	0.0050	Fam1, Fam17, Fam27, Fam32	24.1	4 out of 6
*DIS3L2*	NM_001257281.1:c.1651_1652insGGG	NP_001244210.1:p.Glu550_Ala551insGly	SNP	Missense	0.0022	0	Fam17, Fam26, Fam3	11.4	NA
*PPP6R2*	NM_001242898.1:c.1421G > A	NP_001229827.1:p.Arg474His	SNP	Missense	0.0049	0.0076	Fam1, Fam2, Fam35	23.5	4 out of 6
*PPP6R2*	NM_001242898.1:c.2345G > A	NP_001229827.1:p.Gly782Asp	SNP	Missense	0.0049	0.0094	Fam1, Fam2, Fam35	14.8	2 out of 6
*TM4SF19*	NM_001204897.1:c.586A > G	NP_001191826.1:p.Thr196Ala	SNP	Missense	0.0043	0.0066	Fam19, Fam32, Fam4	22.5	3 out of 6

Next, we searched the literature for genes already associated with DDH in humans and in dogs, as canine hip dysplasia is a relevant natural model for the disease (Pascual-Garrido et al. 2018), and compared it to our data. The complete list of known association genes and the comparison are shown in Online Resource 6. We found novel variants in reported DDH candidate genes in humans: *CDH7*, *CX3CR1*, *DACH1*, *ESR1*, *MYH10*, *NOTCH2*, *POLE*, *TBX4*, and *TENM3*; and in the following genes described in dogs: *COL6A3*, *EVC2*, *INPP5D*, *LAMA2*, *NWD1*, *OTOG, PHF2*, and *SHC3*.

Analysis of CNVs did not detect any common structural variants in regions involving known DDH candidate genes (Online Resource 7). Only one CNV variant was detected in multiple families (family 1 and family 22), and it involved the duplication of exons 19 to 22 of the *ANKRD24* gene.

### Functional analysis of the variants and candidate genes

To identify the most deleterious variants that may cause DDH in our cohort, we used in silico predictions to filter and select the most damaging, rare, and relevant variants. We included shared variants within families and used a more stringent filtering for variant frequency in the total population. We selected only variants with a MAF equal or under 0.01, and less than 5 homozygotes as before, but also included less than 590 alternate allele counts in the general population, which would be the expected number assuming an autosomal dominant mode of inheritance and an average DDH incidence of 1:3400. Variants also either had to have a loss-of-function (LoF) mutation, or a very high prediction score with 5 or 6 algorithms predicting deleteriousness, or a CADD score of over 25. Among the identified 2640 rare and shared variants from our study group, there were 722 of them predicted to be highly damaging (the complete list of the most damaging variants can be found in Online Resource 8).

**Fig. 3:**
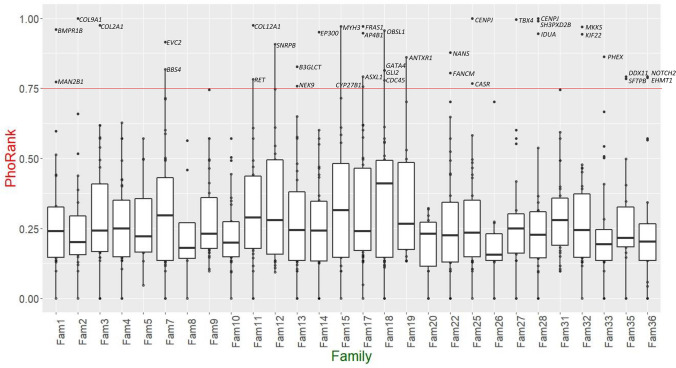
DDH relevant genes with the most damaging mutations. In the figure is shown a box-plot of the 722 most damaging variants and their distribution across the families. The most damaging variants found in the affected individuals from 28 families were selected based on a high prediction of deleteriousness. The variants were either loss-of-function (LoF), had a CADD score of over 25, or were predicted to be damaging by 5 or more in silico tools. The box-plot shows the distribution of PhoRank values of the variants across the families. A PhoRank of over 0.75 predicts a related function of the gene respect to hip dysplasia phenotype using Gene Ontology (GO) and Human Phenotype Ontology (HPO). The median line shows the median, the hinges are the first and third quartiles, and the whiskers extend to the largest and smallest value, but not further than 1.5*IQR (first and third interquartile range) from the hinge. The names of the genes carrying very damaging variants and a PhoRank score of over 0.75 are visible over the red line

As most of the families still had many very damaging variants, we used the PhoRank algorithm in an attempt to select for the most relevant ones. The PhoRank algorithm assigns a score, from 0 to 1, to each gene according to closeness to a user-specified phenotype (hip dysplasia in this case) and it is based on the GO and the HPO ontology. The distribution of very damaging variants across the families and according to PhoRank is visualized in Fig. 3. For most of the variants the median value of PhoRank was low (under 0.325). However, few variants had a very high score: 36 genes carrying 37 very damaging variants had a PhoRank score over 0.75, indicating closeness to the DDH phenotype (Fig. 3 and Online Resource 9). Among these, known associated genes were *TBX4* and *NOTCH2*. We further found very damaging mutations in three collagen genes: *COL12A1*, *COL2A1*, and *COL9A1*. Mutations in *COL2A1* are known to cause spondyloepiphyseal dysplasia and *COL2A1* has previously been related to osteoarthritis secondary to DDH (Granchi et al. 2002). We also identified very damaging variants in three other genes involved in chondrodysplasias: *BMPR1B*, *KIF22*, and *NANS*. A known pathogenic mutation was found in *IDUA*: c.1829-1G > A. ALoF mutation causing mucopolysaccharidosis of type 1 (Cobos et al. 2015), an autosomal recessive disorder that commonly involves the hips, with similar clinical and radiological presentation as DDH (Oussoren et al. 2021).

To conclude, many families presented with more than one very damaging variant present in all affected members, leading to the assumption of a multiple genetic contribution to the phenotype. As many families did not present damaging variants with high PhoRank, it is possible that genes not yet associated to the disease are actually relevant in these cases.

### Mutational burden analysis

In an attempt to investigate which genes are the most relevant, we decided to look for those genes that are most frequently affected when considering all families together. As previously, we filtered for shared variants between the affected members within the families and low variant frequency in the general and Danish population, and then we selected for genes carrying variants in more than one family. We found 322 genes to be affected in at least two families, carrying a total of 663 variants (Table 2).

**Table 2:** Number of genes bearing rare variants found in more than one family

Genes common to	Number of genes
9 families	1
5 families	4
4 families	11
3 families	40
2 families	266
Total	322

**Fig. 4:**
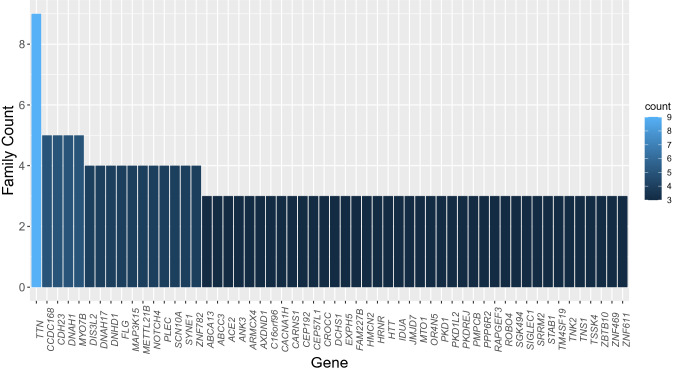
The most frequently affected genes in our study. Histogram with the 56 genes bearing rare variants in multiple DDH families and the respective family count. *TTN* is the most affected gene occurring with variants in nine families. For simplicity, only the genes having variants in three or more families are shown

Titin (*TTN*) was the most affected gene in our study group, as we found nine families and eleven missense variants in the *TTN* gene, with one family carrying two variants. Next in line were genes having variants in 5 families and included *CCDC168*, cadherin 23 (*CDH23*), *DNAH1*, and *MYO7B*. Figure 4 shows a list of the 56 most commonly hit genes as they were found carrying variants in three or more families.

Since our most affected gene (*TTN*) is also the longest in the genome, we ran a correlation test between gene length (considering only the 5’ UTR, the 3’UTR, and the cds) and the number of variants, and gene length and the number of families with variants for each gene. A correlation coefficient of *r* = 0.42 and *r* = 0.36, respectively, suggested that gene length by itself was not a relevant factor in determining how many variants we would find, or how many families would carry the variants.

### Biological function and candidate pathways

To investigate whether the affected genes cluster in defined pathways, we performed GO term enrichment and pathway analysis taking into consideration only genes affected in two or more families.

**Fig. 5:**
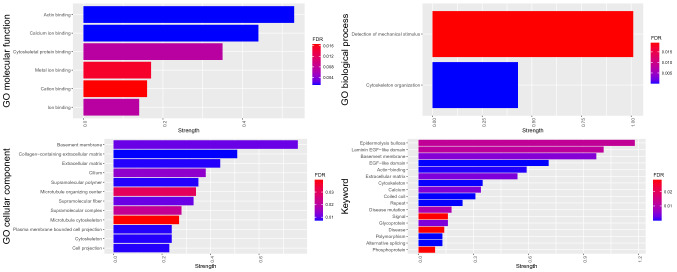
GO term analysis of the most affected genes in our study. In the figure is shown an enrichment analysis made with STRING (string-db.org) of the GO terms within molecular function, biological process, cellular component, and Keyword analysis taking into consideration 322 genes carrying variants in two or more families. The strength describes the enrichment effect and it is measured as Log10 (observed / expected). The ratio is between the number of observed genes from our network in a specific category, and the expected number of genes falling in the same category from a random network of the same size. False discovery rates (FDR) are p-values corrected for multiple testing using the Benjamini–Hochberg procedure. Enrichments with an FDR of under 0.05 are considered significant and displayed in the figure

The number one hit in the analysis of the enrichment of GO biological process terms was detection of mechanical stimulus followed by cytoskeleton organization (Fig. 5 and Online Resource 10). Among other significantly enriched GO terms there were many belonging to the extracellular matrix, microtubules, or the cytoskeleton topology and function. More precisely, enriched GO cellular component terms were the basement membrane and extracellular matrix, the cilium, and microtubule cytoskeleton. While enriched GO molecular function terms were actin binding, calcium ion binding, and cytoskeletal protein binding.

Significantly enriched keywords associated with our group of genes were Epidermolysis bullosa due to four genes with variants in our study group (*EXPH5*, *PLEC*, *COL7A1*, *LAMB3*). Other terms were already mentioned in the GO term enrichment such as laminins, the basement membrane, actin binding, the extracellular matrix, and the cytoskeleton.

### Protein interaction analysis

**Fig. 6:**
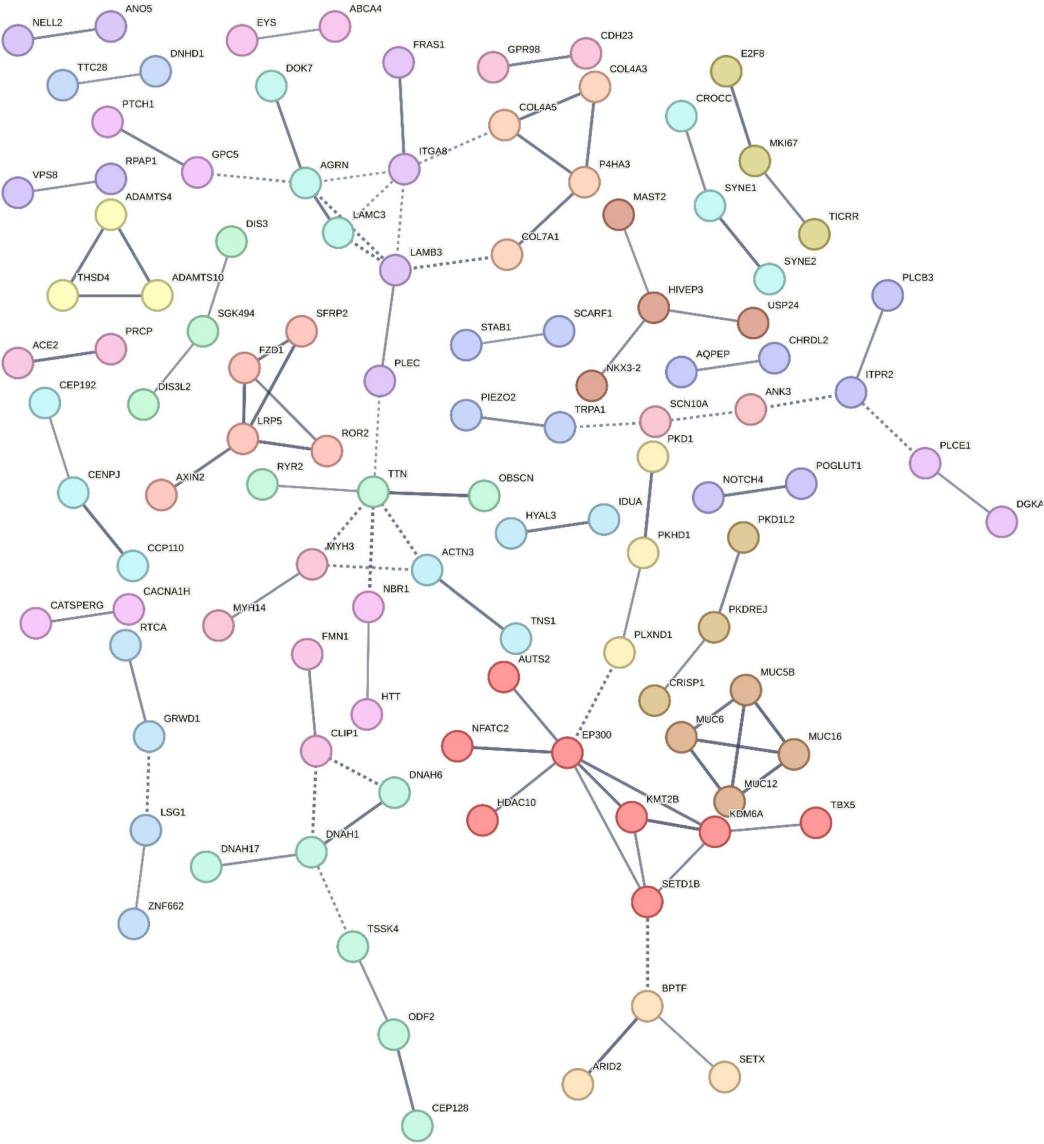
Protein interaction network of the most frequently affected genes**.** In the figure is shown a protein interaction network calculated using the STRING software. The spherical nodes in the network are single proteins; loose nodes with no connection to other proteins are not visualized in this representation. The lines represent functional and physical protein associations, while the thickness of the lines represents the confidence level, with the thickest representing maximum confidence. The different colors represent different clusters of proteins

To investigate which protein networks the identified genes belonged to, we used STRING (https://string-db.org/) and generated a protein interaction network using as input the list of 322 genes affected in at least two families, using a required high confidence interaction score of 0.7. The resulting protein network was rather large (Fig. 6, Online Resource 11 for high resolution), with 8 clusters among which the biggest cluster had EP300, a transcriptional co-activator that regulates cell proliferation and differentiation, and several histone deacetylases and methyl-altering enzymes (Fig. 6 and Online Resource 10, MCL clustering tab). The second cluster in size was formed around AXIN-2, an inhibitor of the Wnt signaling pathway.

In the overall, the resulting protein interaction network had significantly more interconnections than expected (100 vs 48 expected), as the protein interaction network enrichment *p* value was 2.87e-11. The value was calculated for 320 number of nodes (proteins) in the network, and (Markov Cluster algorithm) MCL clustering was used with an inflation parameter of 3. Overall, this indicates that the genes from our list form a larger group of biologically connected proteins than what a random set of genes of the same dimensions would make.

## Discussion

DDH is a common musculoskeletal condition influenced by genetic, mechanical, and environmental factors, resulting in a wide range of phenotypic manifestations. The earlier DDH is diagnosed, the simpler and more effective are the treatments. However, late detection of instable hips is a persistent problem, and the phenotypic heterogeneity of DDH poses a serious challenge for diagnostics, especially in adults (American Academy of Pediatrics 2000; Harsanyi et al. 2020b). Predictive genetic testing may improve significantly diagnostic methods and patient management in families with a positive family history of DDH.

Until now, 34 genes have been related to DDH in humans (Mabuchi et al. 2006; Kapoor et al. 2007; Dai et al. 2008; Rouault et al. 2010; Wang et al. 2010; Shi et al. 2011; Jia et al. 2012; Tian et al. 2012; Feldman et al. 2013, 2019; Sekimoto et al. 2013; Tuzovic et al. 2013; Zhao et al. 2013, 2017; Hao et al. 2014; Liu et al. 2014; Cengic et al. 2015; Sun et al. 2015; Watson et al. 2015; Basit et al. 2017, 2018; Li et al. 2017; Ma et al. 2017; Qiao et al. 2017; Hatzikotoulas et al. 2018; Sadat-Ali et al. 2018; Zhang et al. 2018; Zhu et al. 2019; Harsanyi et al. 2020a; Kenanidis et al. 2020), sometimes with contradictory results (Rubini et al. 2008). However, no tight phenotype–genotype correlations have been established and the molecular mechanisms of the disease have just started to be elucidated. To this end, canine hip dysplasia is a relevant natural animal model for DDH (Pascual-Garrido et al. 2018). Studies focusing on genetic markers for hip dysplasia in dogs have unravelled over 50 candidate genes, with little overlap with human candidate genes (Zhou et al. 2010; Pfahler and Distl 2012; Fels and Distl 2014; Fels et al. 2014; Lavrijsen et al. 2014; Sanchez-Molano et al. 2014; Ginja et al. 2015). It is generally thought that sequence variants in individual genes are the cause of DDH, although a simple single-gene inheritance model is very unlikely to be able to explain the whole complexity of the phenotypic and genetic heterogeneity in DDH. A multiple-gene model of inheritance was already proposed in an initial study (Wynne-Davies 1970). A two-gene model identified by exome sequencing has been proposed for a family carrying *HSPG2* and *ATP2B4* variants (Basit et al. 2018). As modern sequencing techniques allow now for genome-wide investigations, several studies using next generation sequencing reported even more loci/genes in relation to DDH. Zhu et al. used genome sequencing and reported 1344 DDH associated-genes from a study including ten patients and ten unaffected relatives (Zhu et al. 2019), while Yan et al. (2019) performed a GWAS in a cohort of Chinese patients reporting 406 DDH-associated genes.

We report exome sequencing results from 28 families, with at least two members affected by DDH, where we also observe a high number of candidate genes, with 322 genes occurring with variants in multiple families. Previously described genes *DACH1*, *MYH10*, *NOTCH2, TBX4, EVC2*, *OTOG*, and *SHC3* have highly damaging variants also in our study group; of these *TBX4, EVC2*, *NWD1*, and *OTOG* have variants in more than one family further strengthening the notion that they are involved in the etiopathogenesis of DDH.

Among novel genes, four genes stand-out from our analysis as they carry the same variants in four (*METTL21B*), and three (*DIS3L2*, *PPP6R2*, and *TM4SF19*) families. We identify also *TTN*, *CDH23*, *CCDC168*, *DNAH1*, and *MYO7B* as the most affected genes, presenting with variants in nine (*TTN*) and five families. Although neither of these genes have been were previously related to DDH, Zhu et al. (2019) lists *TTN*, *MYO7B*, and *DIS3L2* among the DDH-specific genes in their study group, while *CDH23* was associated to DDH in the GWAS study from Yan et al. (2019).

If we considered groups of similar genes, the number of families with variants would increase considerably. For example, *NOTCH2*, which encodes a transmembrane surface receptor, together with other three Notch receptors plays a fundamental role in bone morphogenesis. Besides a family carrying variants in the known DDH candidate gene *NOTCH2*, we also found four families carrying variants in *NOTCH4,* and one family with a variant in *NOTCH3*. Similarly, we found two missense variants in the known DDH gene *TBX4*, but we also found two families with *TBX5* variants, which is not a known DDH candidate gene. The myosin heavy chain 10 (*MYH10*) gene is another previously reported DDH gene (Tuzovic et al. 2013); we found one family with a very damaging variant in *MYH10*, but also a family with a variant in *MYH7B*, two families in *MYH14,* and two families with variants in *MYH3*. Laminin 2 (*LAMA2*) is a candidate extracellular matrix gene described in hip dysplasia in dogs (Lavrijsen et al. 2014). Besides finding a family with a *LAMA2* variant, we also found a family with a *LAMA1* variant, and one family with a *LAMA3* variant.

We applied pathway analysis to provide insights into which groups of genes are the most commonly affected. As expected, we found the basement membrane, the extracellular matrix, and the cilium among enriched cellular components, while ion-binding and cytoskeletal protein binding among enriched functions. All these elements participate in the detection of the mechanical stimulus, which is another enriched process in our analysis.

Our pathway analysis is in line with previous results (Yan et al. 2019; Zhu et al. 2019) as other authors found ion and calcium binding, the cytoskeleton, and the microtubules as enriched categories, similarly to us.

We hypothesize that the molecular mechanism of DDH can be traced back to a dysfunction at some level of the transduction of the mechanical stimulus, either in the extracellular matrix, at the level of the cell membrane, the cytoplasm, or in gene activation in the nucleus.

## Conclusions

We identified four novel DDH candidate genes occurring with variants in several different families, and we found very damaging variants in the following known candidate genes from human and canine hip dysplasia: *DACH1*, *MYH10*, *NOTCH2, TBX4, EVC2*, *OTOG*, and *SHC3*, which has important implications for the development of diagnostic methods for DDH. Our study also supports the hypothesis that multiple genes contribute to the development of DDH, as we found many families with several damaging variants shared between the affected members. However, further studies are needed to elucidate their role in the development of the DDH phenotype. We conclude that DDH is a polygenic disorder, and we propose that impaired transduction of the mechanical stimulus is involved in the pathophysiological mechanism of the disease.

## Supplementary Information

Below is the link to the electronic supplementary material.

Supplementary file1 (PDF 324 KB)

Supplementary file2(DOCX 9935 KB)

Supplementary file3 (XLSX 28 KB)

Supplementary file4 (XLSX 331 KB)

Supplementary file5 (XLSX 10 KB)

Supplementary file6 (XLSX 18 KB)

Supplementary file7 (XLSX 10 KB)

Supplementary file8 (XLSX 94 KB)

Supplementary file9 (XLSX 22 KB)

Supplementary file10 (XLSX 46 KB)

Supplementary file11 (PDF 315 KB)

## Data Availability

In order to comply with the ethical approval, anonymized data supporting the findings of this study is available within the main article and its supplementary data, containing all the rare and shared variants within the families. Raw sequencing data are not available for broad data sharing because of the ethical and legal restrictions on data usage, since the patients did not provide informed consent that covers deposition to public repositories, sharing, and additional use of their genetic data. Please contact the corresponding author (Maja Dembic, Maja.Dembic@rsyd.dk), if you would like to request the raw data; it will, however, require an additional application to the ethical committee. All software used is described and cited throughout the manuscript. Custom scripts are available at https://github.com/MaDemb/DDH.
