# Supplementary material for: Whole exome sequencing of 28 families of Danish descent reveals novel candidate genes and pathways in developmental dysplasia of the hip
Source: Mol Genet Genomics. 2022 Dec 1;298(2):329–42. doi: 10.1007/s00438-022-01980-5 (PMC9938029; doi:10.1007/s00438-022-01980-5)
Supplement: Supplementary file 1 — Supplementary file1 (PDF 324 KB) [file 438_2022_1980_MOESM1_ESM.pdf]

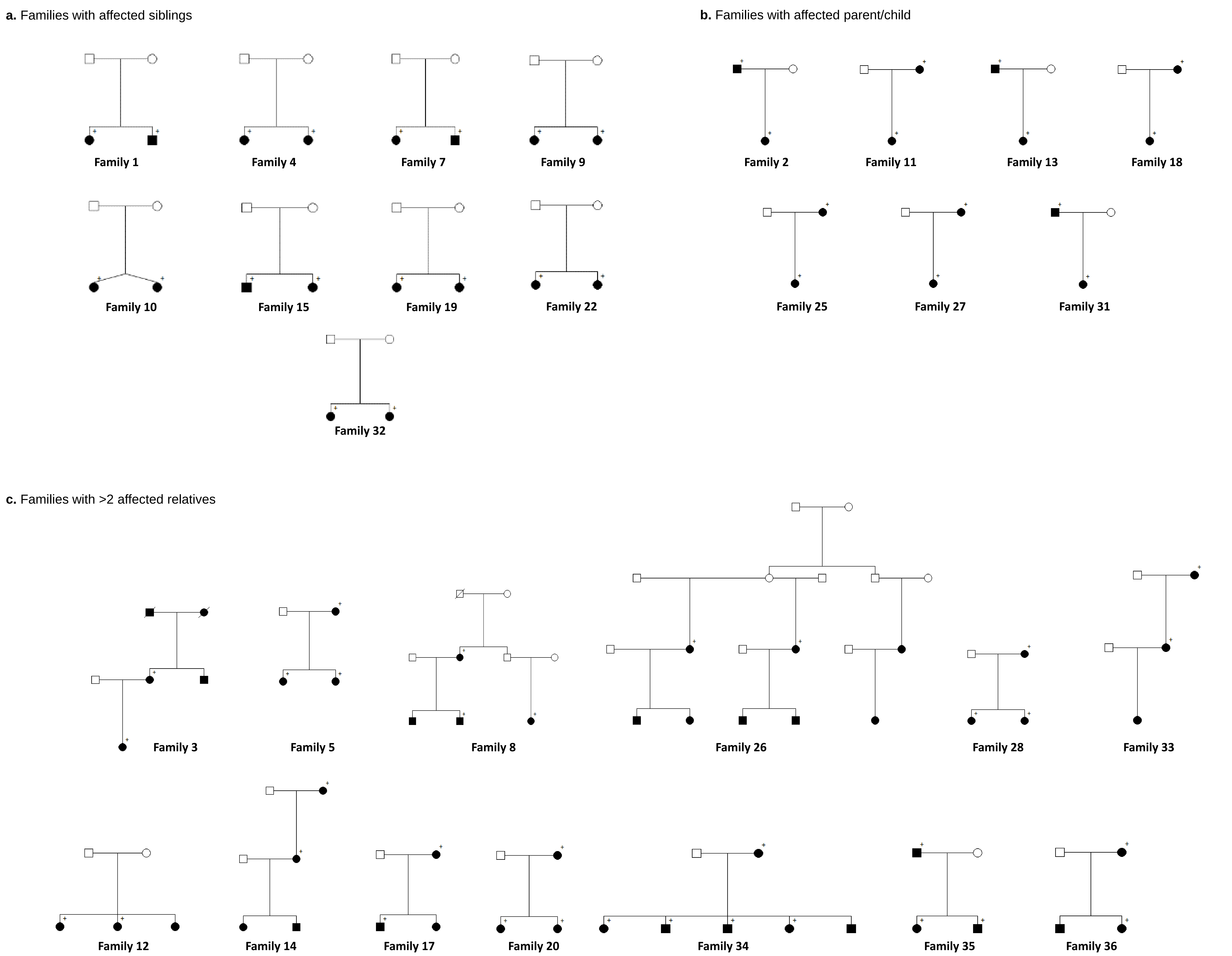

Supplementary Fig. 1 Pedigrees of the 29 families included in the study

(a) Families with affected siblings (N=9), (b) families with affected parent/child (N=7), and (c) families with more than two affected relatives (N=13). Affected persons marked with a ”+” are included in the study.
