# Supplementary material for: Whole exome sequencing of 28 families of Danish descent reveals novel candidate genes and pathways in developmental dysplasia of the hip
Source: Mol Genet Genomics. 2022 Dec 1;298(2):329–42. doi: 10.1007/s00438-022-01980-5 (PMC9938029; doi:10.1007/s00438-022-01980-5)
Supplement: Supplementary file 2 — Supplementary file2(DOCX 9935 KB) [file 438_2022_1980_MOESM2_ESM.docx]

**Supplementary material**

**Whole exome sequencing of 28 families of Danish descent reveals novel candidate genes and pathways in developmental dysplasia of the hip**

Maja Dembic*^1,2,3^, Lars van Brakel Andersen^1,3^, Martin Jakob Larsen^1,3^, Inger Mechlenburg^4,5^, Kjeld Søballe^4,5^, Jens Michael Hertz^1,3^

^1^Department of Clinical Genetics, Odense University Hospital, J. B. Winsløws Vej 4, 5000, Odense C, Denmark.

^2^Department of Mathematics and Computer Science (IMADA), University of Southern Denmark, Campusvej 55, 5230 Odense M, Denmark.

^3^Department of Clinical Research, University of Southern Denmark, Winsløwparken 19, 5000 Odense C, Denmark.

^4^Department of Orthopedic Surgery, Aarhus University Hospital, Palle Juul-Jensens Boulevard 99, 8200 Aarhus N, Denmark.

^5^Department of Clinical Medicine, Aarhus University, Palle Juul-Jensens Boulevard 82, 8200 Aarhus N, Denmark.

***Corresponding author:**

Maja DembicMaja.Dembic@rsyd.dk, Department of Clinical Genetics, Odense University Hospital, J.B. Winsløws Vej 4, 5000 Odense C, Denmark; ORCID: 000-0003-2505-6863.

**Supplementary Fig.1 Variant allele frequency plots of all sequenced samples**





Variant allele frequency values (VAF) in the sequencing reads were plotted as a quality measure. Homozygote variants have a VAF of 1, while hetorozygote variants have a VAF around 0.5. When there is cross-contamination, homozygote variants from another sample will appear as variants with intermediate VAF values as they will be mixed with reads from another sample that may not harbor that variant. Other quality issues regarding samples may also be visible. All our samples have an expected VAF plot, except sample A5B3 from family 28.


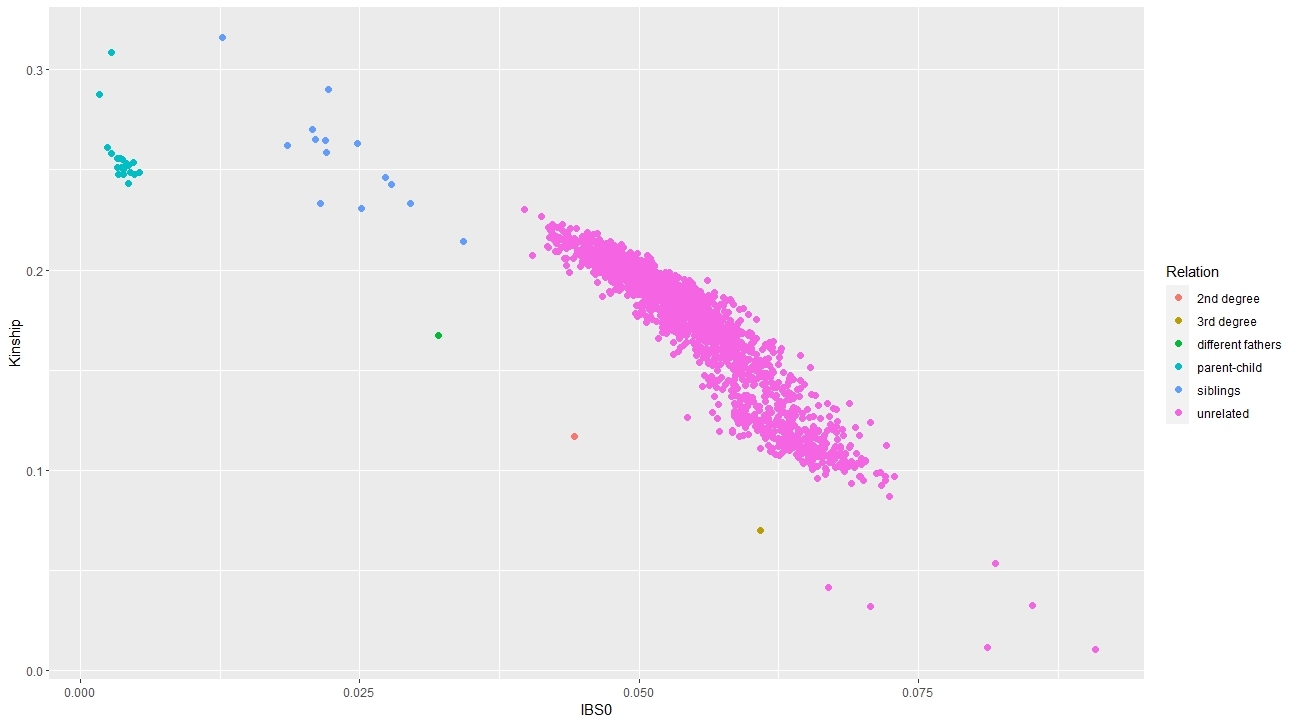


**Supplementary Fig.2 Kinship analysis diagram of 66 patients included in our study**

In the diagram are plotted the values obtained for Kinship and identity by similarity (IBS0) as obtained by the PLINK software for the pairwise analysis of samples. A distanced group with the highest kinship and lowest IBS0 values represents the group of all sibling relations. A second more distanced group on the x-axis represents the parent-child group, while further down on the x-axis is the group of most un-related individuals. Three points stand-out as separate from these two groups and under the unrelated group, the first one representing two half-siblings (having two different fathers). The other two out-layers represent a 2^nd^ degree and a 3^rd^ degree relatives that do not fit the rest of the unrelated group.


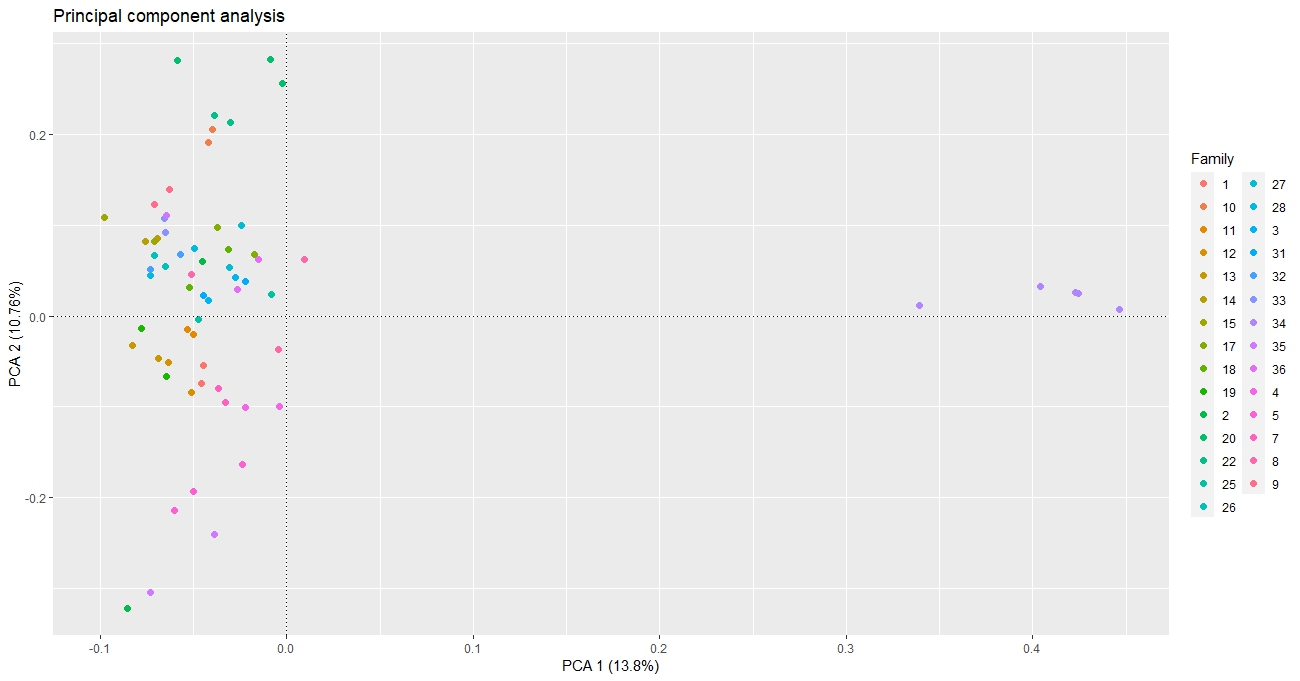


**Supplementary Fig.3 PCA analysis of the 66 patients and 29 families initially included in the study**

Plot of the first two component in the PCA analysis shows that all five members of the family 34 are grouped together more distantly from the rest of the individuals. None of the remaining individuals form an distinct group within a family and position themselves so far away from the rest. Some variability between individuals is observed in the PCA2 (y-axis), which most probably reflect normal individual differences within a homogenous population.
