# Supplementary figures and images for: Whole exome sequencing of 28 families of Danish descent reveals novel candidate genes and pathways in developmental dysplasia of the hip
Source: Mol Genet Genomics. 2022 Dec 1;298(2):329–42. doi: 10.1007/s00438-022-01980-5 (PMC9938029; doi:10.1007/s00438-022-01980-5)

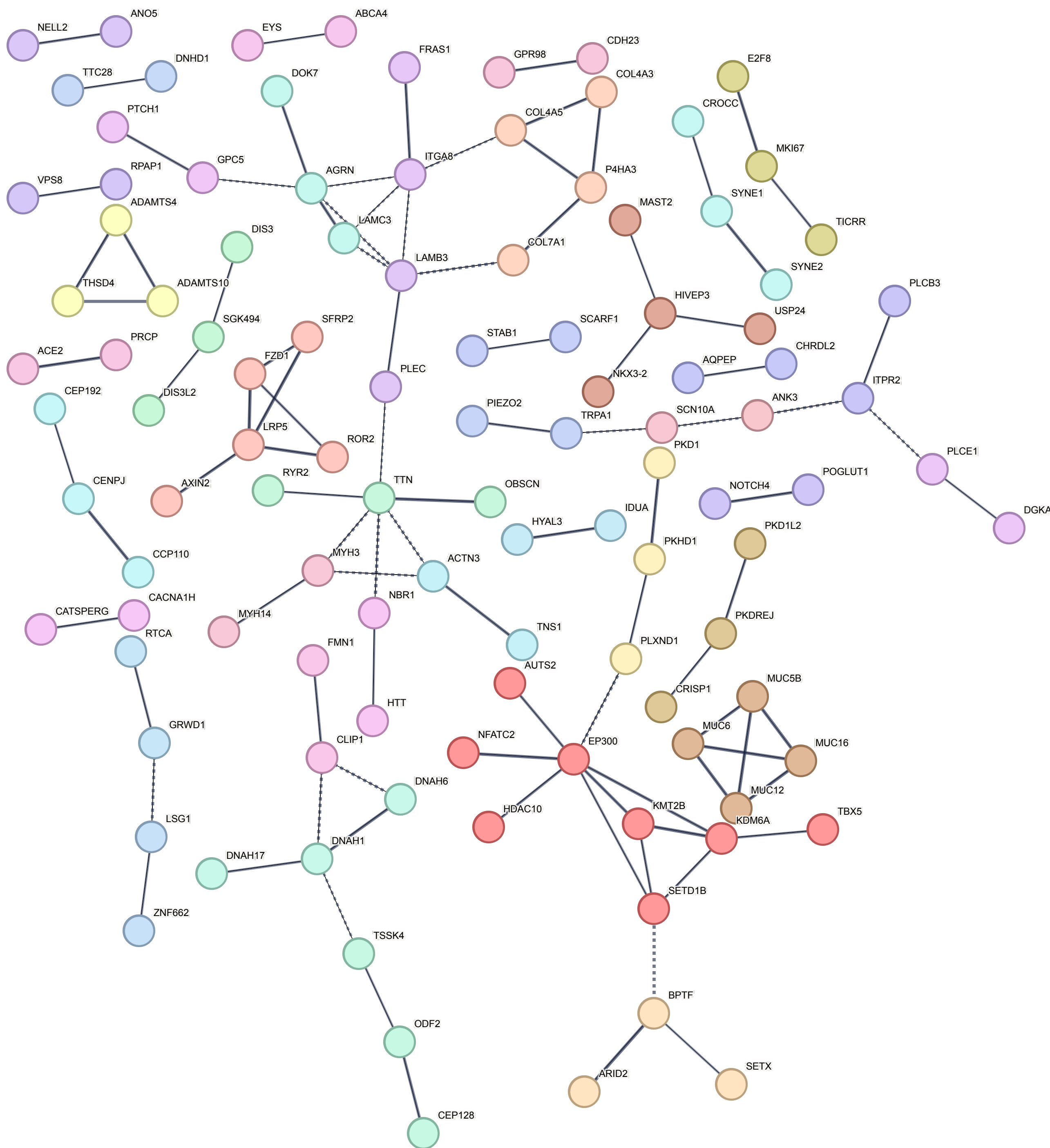

Supplement: Supplementary file 11 — Supplementary file11 (PDF 315 KB) [file 438_2022_1980_MOESM11_ESM.pdf]
